# A hierarchical expert-guided machine learning framework for clinical decision support systems: an application to traumatic brain injury prognostication

**DOI:** 10.1038/s41746-021-00445-0

**Published:** 2021-05-07

**Authors:** Negar Farzaneh, Craig A. Williamson, Jonathan Gryak, Kayvan Najarian

**Affiliations:** 1 Department of Computational Medicine and Bioinformatics, University of Michigan, Ann Arbor, MI USA; 2 Michigan Center for Integrative Research in Critical Care, University of Michigan, Ann Arbor, MI USA; 3 Department of Neurological Surgery, University of Michigan, Ann Arbor, MI USA; 4 Department of Neurology, University of Michigan, Ann Arbor, MI USA; 5 Michigan Institute for Data Science, University of Michigan, Ann Arbor, MI USA; 6 Department of Emergency Medicine, University of Michigan, Ann Arbor, MI USA; 7 Department of Electrical Engineering and Computer Science, University of Michigan, Ann Arbor, MI USA

**Keywords:** Risk factors, Disease-free survival, Brain injuries, Outcomes research

## Abstract

Prognosis of the long-term functional outcome of traumatic brain injury is essential for personalized management of that injury. Nonetheless, accurate prediction remains unavailable. Although machine learning has shown promise in many fields, including medical diagnosis and prognosis, such models are rarely deployed in real-world settings due to a lack of transparency and trustworthiness. To address these drawbacks, we propose a machine learning-based framework that is explainable and aligns with clinical domain knowledge. To build such a framework, additional layers of statistical inference and human expert validation are added to the model, which ensures the predicted risk score’s trustworthiness. Using 831 patients with moderate or severe traumatic brain injury to build a model using the proposed framework, an area under the receiver operating characteristic curve (AUC) and accuracy of 0.8085 and 0.7488 were achieved, respectively, in determining which patients will experience poor functional outcomes. The performance of the machine learning classifier is not adversely affected by the imposition of statistical and domain knowledge “checks and balances”. Finally, through a case study, we demonstrate how the decision made by a model might be biased if it is not audited carefully.

## Introduction

Traumatic Brain Injury (TBI), often referred to as the “silent epidemic”, is the leading cause of death among young Americans^1,2^. While accurate early prognostication of TBI outcomes can guide physicians and families through early resuscitation and treatment planning, such a prognostic system remains unavailable. After initial resuscitation, most patients with severe brain injury die as a result of withdrawal of life-sustaining treatment. Consequently, there is a critical need for accurate tools that can identify and prevent early withdrawal from treatment in severe TBI patients who still have a reasonable chance for a favorable outcome^3,4^. Even experienced neurosurgeons and neurocritical care practitioners frequently overestimate the likelihood of poor neurological outcome in comparison with validated prediction scores^5^. By accurately predicting long-term functional outcomes, physicians can make more evidence-based and informed decisions in such cases.

Over the last four decades, several studies aimed to produce prognostic models by using patient responsiveness^6–8^, radiographic images^9–11^, or said images in combination with other risk factors^11–16^. However, the accuracy and generalizability of these models over complex and heterogeneous cohorts are questionable^17,18^. A primary reason for the failure of these models is the oversimplification of the risk assessment method employed, in which only a limited number risk factors are considered.

Artificial intelligence has shown great promise in enhancing the medical decision-making process, specifically when there is a significant complexity and uncertainty involved with the risk assessment task^19^. Machine learning algorithms enable integrating multiple sources of information in a complex non-linear fashion for accurate data-informed prognostication. A few recent studies sought to tackle the oversimplification in previous TBI prognosis studies by employing machine learning methods^20,21^. However, this approach comes with a trade-off: a sophisticated machine learning model’s rationale for an individual decision is not readily interpretable by clinicians. The black box nature of such algorithms prevents them from being integrated into medical practice where transparency is imperative^22–26^. Acceptance of such models by clinicians in real-world settings requires the underlying reasoning of a model to be explainable, understandable, and trustworthy^25–27^.

Another concern is the susceptibility of machine learning models to poor performance over unobserved data. This is particularly acute in medical applications where, due to privacy and intellectual property issues, it is costly and often impractical to have an ideal data set that is sufficiently large and heterogeneous to represent all subtypes of the condition under study. Thus, during the training stage, machine learning can potentially learn unrealistic cohort-specific patterns that are not generalizable^25^ or clinically significant. Such models introduce additional sources of bias to the prediction model^25^, which will not be readily detectable if employed in a black box fashion.

In this work, we propose a machine learning framework that incorporates additional layers of statistical inference and human expert validation to create an *intelligible model* for predicting long-term functional outcomes of TBI patients using data available at the time of hospital admission. Inspired by Caruana et al.^27^, an intelligible model is defined as a model that is both interpretable and aligned with clinical domain knowledge. The proposed machine learning framework constructs an intelligible model through performance explanation, human expert validation, and final model training. To explain the decision-making process of the machine learning model, Shapley values were used to estimate the contribution of each variable to the final decision^28,29^. Next, the contribution of each variable was clinically validated at the population level, with variables determined to be non-robust or exhibiting counterintuitive behaviors subsequently excluded. The results of this process suggest that including counterintuitive features introduces bias to the model. To further explore this hypothesis, a case study was performed on one of the features with counterintuitive behavior in which its impact on model bias was analyzed.

## Results

### Study cohort

In this study, as in most recent TBI clinical trials, the long-term functional outcome after TBI is assessed using the Glasgow Outcome Scale-Extended (GOSE), a global scale for functional outcomes, at 6 months after injury. The original Glasgow Outcome Scale (GOS) and its more detailed and recent revision, the GOSE, are the most widely accepted systems to rate TBI outcomes, having been used in more than 90% of high-quality TBI randomized trials. The GOSE has been extensively validated, is the most widely cited measure of acute brain injury outcomes, and is recommended by both the US National Institutes of Health (NIH) and the UK Department of Health^30^. In this study, GOSE 1–4 (death, persistent vegetative state, and severe disability) were regarded as unfavorable outcomes, while GOSE 5-8 (moderate disability, and good recovery) correspond to patients with favorable outcomes.

This is a secondary analysis of the Progesterone for Traumatic Brain Injury Experimental Clinical Treatment (ProTECT) III data set that includes adults who experienced a moderate to severe brain injury caused by blunt trauma (ClinicalTrials.gov identifier NCT00822900)^31^. This study is approved by the University of Michigan Institutional Review Boards (IRB). The written informed consent from patients is waived by IRB because this study involves no more than minimal risk to the subjects. Patients were excluded from ProTECT III if they had an initial Glasgow Comma Scale (GCS) of 3, bilateral dilated unresponsive pupils, or were otherwise determined to have non-survivable injuries. The data set includes electronic data for 882 patient^31^. Among the 882 patients, 831 met the inclusion criteria. Of 831 individuals admitted to the hospital, 348 were identified to have experienced poor outcomes, with the remaining 483 attaining a favorable recovery at six months.

A rich source of patient-level information is available in the Electronic Health Records (EHR) contained within the ProTECT III data set. This information includes demographic data, baseline features, radiology reports, laboratory values, injury severity scores, and medical history. Demographics and clinical characteristics of the patient cohort are summarized in Table 1. In this study, only data available at the time of hospital admission was used.

**Table 1:** Demographic and clinical characteristics of study subjects.

Characteristic	GOSE ≤ 4	GOSE > 4
Total subjects, *n*	348	483
Female, *n*	97 (27.87%)	127 (26.29%)
Age, median [Q1, Q3]	45 [29, 59]	31 [22, 45]
Abbreviated injury score [Q1, Q3]	29 [22, 36]	22 [14, 29]
Head injury severity score, median [Q1, Q3]	4 [4, 5]	3 [3, 4]
Cause of injury		
Motor vehicle collision, *n*	99 (28.45%)	204 (42.24%)
Motorcycle/scooter/ATV/bicycle crash, *n*	82 (23.56%)	126 (26.09%)
Pedestrian struck by moving vehicle, *n*	66 (18.97%)	42 (8.70%)
Fall, *n*	63 (18.10%)	70 (14.49%)
Assault, *n*	24 (6.90%)	22 (4.55%)
Other or unknown, *n*	14 (4.02%)	19 (3.93%)
Initial Glasgow coma scale		
Motor response, median [Q1, Q3]	4 [3, 5]	5 [4, 5]
Eye opening response, median [Q1, Q3]	1 [1, 2]	2 [1, 3]
Verbal response, median [Q1, Q3]	1 [1, 2]	2 [1, 2]
Radiology findings		
Subdural hematoma, *n*	224 (64.37%)	182 (37.68%)
Subdural hematoma (max width), median [Q1, Q3]	17.5 [0.0, 70.0]	0 [0.0, 23.5]
Subarachnoid hemorrhage (#), median [Q1, Q3]	2 [1, 3]	0 [0, 2]
Intra-ventricular hemorrhage, *n*	114 (32.76%)	75 (15.53%)
Intraparenchymal hematoma (max width), median [Q1, Q3]	0 [0, 0]	0 [0, 0]
Brain contusion (#), median [Q1, Q3]	1 [0, 2]	0 [0, 1]
Brain contusion (max width), median [Q1, Q3]	2.0 [0.0, 38.0]	0.0 [0.0, 13.75]
Diffuse axonal injury finding (#), median [Q1, Q3]	0 [0, 1]	0 [0, 0]
Third ventricle compression, *n*	125 (35.92%)	53 (10.97%)
Transtentorial herniation, *n*	97 (27.87%)	34 (7.04%)
Laboratory values		
Glucose, median [Q1, Q3]	147.0 [126.0, 174.0]	139.0 [115.0, 167.25]
Hgb, median [Q1, Q3]	13.40 [12.15, 14.60]	14.0 [12.70, 15.0]
Platelets, median [Q1, Q3]	240.0 [201.0, 288.75]	236.0 [199.0, 283.0]
aPTT, median [Q1, Q3]	26.8 [24.0, 29.8]	25.8 [23.5, 28.1]
INR, median [Q1, Q3]	1.1 [1.0, 1.2]	1.1 [1.0, 1.17]

### Intelligible variable selection using computational analysis and human validation

**Fig. 1:**
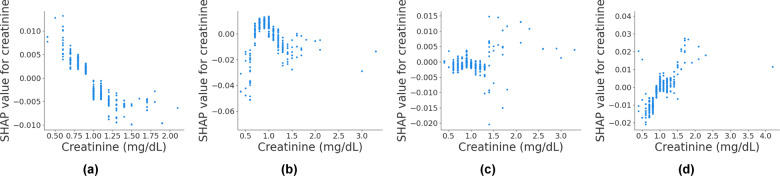
An example of a variable with non-robust contribution behavior. **a**, **b**, **c**, and **d** are the SHAP contribution of creatinine levels to the predicted risk score when training on different bootstrapping sample sets.

Among 62 candidate variables that were extracted from the EHR (see Supplementary Table 1 for the full list of EHR variables and their definitions), 21 were shown to be statistically robust (see Supplementary Table 2 for Kendall’s *τ* correlation coefficients of the robust variables and the corresponding *p-*values). The robustness was estimated with respect to the global distribution of SHAP (SHapley Additive exPlanations) contribution. Of the initial 62 features, the 21 selected features are not necessarily those with the highest contributions. For example, creatinine, WBC, and potassium were among the top features in terms of the amount of contribution; however, their behavior was not statistically robust (Fig. 1 and Supplementary Figs. 1a and 2a).

**Fig. 2:**
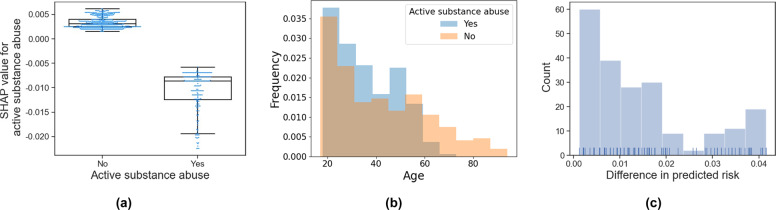
An example of a variable with counterintuitive behavior. **a** Shows that active substance abuse yields a negative contribution. Center line and box limits correspond to median and upper and lower quartiles, respectively. **b** Shows the difference in age distribution between active substance abusers and others. **c** Shows difference in the predicted risk scores of the test population if the active substance abuse value is set zero or one.

The 21 automatically selected robust variables where then carefully evaluated to identify those with unexpected or counterintuitive behaviors (Supplementary Figs. 3 and 4). Three variables were identified by a physician board-certified in neurology and neurocritical care to exhibit behavior contrary to clinical domain knowledge: active substance abuse, inactive gastrointestinal disease, and platelet count (Fig. 2a and Supplementary Fig. 3). The results show that the existence of either active substance abuse or active gastrointestinal disease lowers the risk of poor outcomes, which is contrary to clinical domain knowledge. The decrease in platelet count was observed to be associated with better outcomes which also contradicts medical literature^32,33^. These counterintuitive associations might be either derived from the collinearity between variables, thereby representing an (un)known proxy variable, or induced by noise in the data set, both of which can introduce bias into the model if not addressed properly. The collinearity effect becomes of crucial importance if it exists between these risk factors and the level of care patients received, which can lead to self-reinforcing positive feedback^34^. For example, patients with active gastrointestinal disease, substance abuse, or coagulation dysfunction (low platelet) might receive more aggressive care that affects their outcome. However, if this bias is not accounted for at the algorithm development level, in real-world settings the model will assess these patients as having a lower chance of unfavorable outcome, resulting in them potentially receiving less aggressive care that they would have otherwise.

These biases are studied in the case study on active substance abuse in the following section. Regarding active gastrointestinal disease and platelet count contributions, no meaningful correlation or explanation was observed in the data set. It is possible that they reflect a latent variable or the observed behavior is merely specific to the study cohort.

These three variables were excluded from the study, leaving 18 EHR variables to be included in the final prognostic model. The selected variables include features from radiology reports, laboratory values, and baseline clinical features (see Supplementary Table 1 for detailed information).

**Fig. 3:**
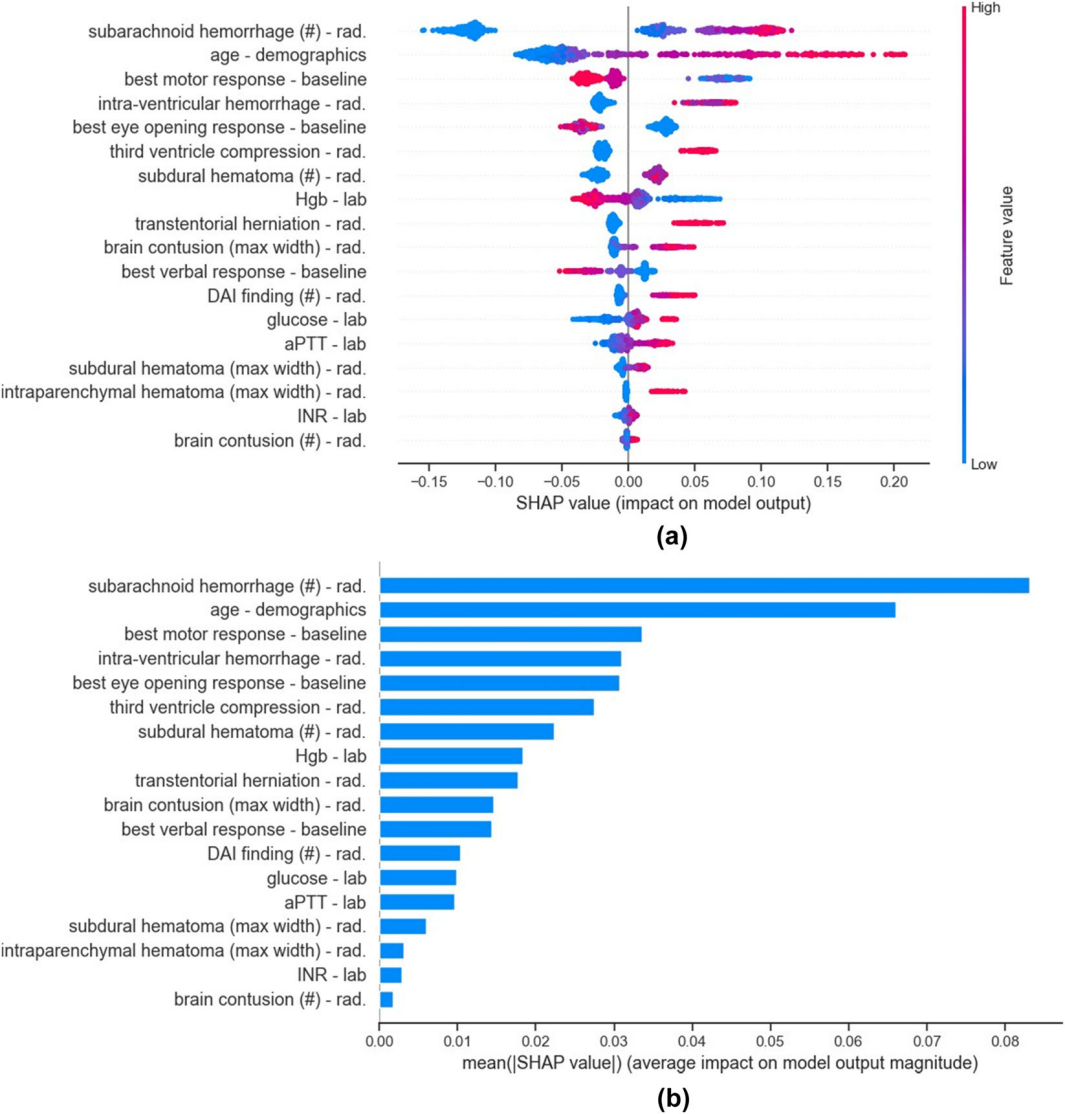
Summary of the SHAP contributions in the final model. **a** Shows the summary plot of the contribution of all 18 variables in the final model. Each point corresponds to one patient, while color corresponds to the value of the variable, with the spectrum from blue to pink associated with low to high values. **b** Shows variables in order of their importance, where importance of a variable is defined by the average of the absolute SHAP values. Variable types are denoted as *rad*: radiology report and *lab*: laboratory value.

### Case study: active substance abuse

In this section, the contribution of active substance (alcohol and non-prescribed drug) abuse to risk prediction, along with its possible underlying explanations and potential concerns if not properly addressed, is evaluated. As shown in Fig. 2a, active substance abuse negatively contributes to the predicted risk of poor outcome, which is counterintuitive as alcohol and substance abuse are independent predictors of mortality risk.

However, in the study data set, patients with active substance abuse tend to be younger (Fig. 2b); thus, this variable might reflect the age of a patient. Moreover, the active substance abuse value is correlated with injury etiology, being more common in patients experiencing TBI due to assault (*ρ* = 0.19, *p*-value < 0.01), and head ISS (Injury Severity Score) (*ρ* = *−*0.12, *p-*value < 0.01). Although no causal correlation can be drawn, we speculate that in the proposed model active substance abuse is a confounding variable due to its simultaneous association with injury severity and assault.

To quantify the effect of active substance abuse on the final prediction, an experiment was performed in which the active substance abuse value was manually set to either zero or one for each test patient. The difference in the predicted risk *δ p* is calculated as

1 $$\delta p_i = \hat p\left( {y_i = 1\left| {X_{i,i \neq a},X_a = 0} \right.} \right) - \hat p\left( {y_i = 1\left| {X_{i,i \neq a },X_a = 1} \right.} \right),$$

where *y*_*i*_ corresponds to the outcome while *X*_*i*_ and *X*_*a*_ are the complete variable set and active substance abuse variable, respectively. Figure. 2c shows the histogram of the difference in the predicted risk on the test data set. The average, minimum, and maximum value of *δ p* are 0.014, 0.001, and 0.042, respectively. Based on these results, it can be concluded that in a scenario in which two TBI patients are admitted to a hospital with identical characteristics except for substance abuse, the patient with substance abuse would be predicted to have on average 1.4% (and up to 4.2%) higher chance of a favorable outcome. To address such collinearity-induced biases, variables that exhibited counterintuitive contribution behaviors were excluded from the variable set.

### Prognosis of traumatic brain injury functional outcome

An XGBoost classifier was implemented to predict the functional outcome - GOSE at 6 months. More information regarding the selection of the XGBoost algorithm is available in Supplementary Methods Section and Supplementary Table 3. The classifier was trained, validated, and tested once using the full set of 62 candidate variable, once using 21 identified robust variables, and once using the final 18 intelligible variables (Table 2). Although the performance on the training set decreased slightly after excluding non-robust and counterintuitive variables; AUC, accuracy, and F1 score performance on the validation and test sets were well-preserved throughout this process. These results support the conclusion that the excluded features do not affect the performance of the model. The proposed model’s predictive performance before and after excluding non-robust and counterintuitive variables is compared with those of other classifiers in the Supplementary Results Section as well as Supplementary Tables 3 and 4.

**Table 2:** Performance of the TBI prognostic model.

	All candidate variables			Excluding non-robust variables			Excluding non-robust & counterintuitive variables		
Sample set	Training	Validation	Test	Training	Validation	Test	Training	Validation	Test
AUC (SD)	0.9372 (0.0236)	0.7822 (0.0126)	0.8094	0.9080 (0.0249)	0.7877 (0.0177)	0.8046	0.8912 (0.0252)	0.7836 (0.0189)	0.8085
Accuracy (SD)	0.8522 (0.0327)	0.7500 (0.0169)	0.7536	0.8165 (0.0317)	0.7484 (0.0250)	0.7440	0.8053 (0.0285)	0.7451 (0.0255)	0.7488
F1 score (SD)	0.8281 (0.0360)	0.7129 (0.0190)	0.7052	0.7855 (0.0344)	0.7104 (0.0299)	0.6864	0.7740 (0.0375)	0.7076 (0.0315)	0.7045
Sensitivity (SD)	0.8477 (0.0305)	0.7434 (0.0489)	0.7011	0.8008 (0.0319)	0.7394 (0.0527)	0.6667	0.8018 (0.0637)	0.7393 (0.0570)	0.7126
Specificity (SD)	0.8554 (0.0440)	0.7549 (0.0456)	0.7917	0.8279 (0.0450)	0.7549 (0.0439)	0.8000	0.8078 (0.0238)	0.7494 (0.0443)	0.7750
Precision (SD)	0.8106 (0.0529)	0.6880 (0.0330)	0.7093	0.7722 (0.0498)	0.6862 (0.0329)	0.7073	0.7500 (0.0252)	0.6813 (0.0329)	0.6966

Performance of the TBI prognostic model trained using all candidate variables, only robust variables, and robust and clinically validated variables. Standard deviation (SD) is calculated over 5 cross-validation folds.

### Explaining the rationale behind predicted risk scores

**Fig. 4:**
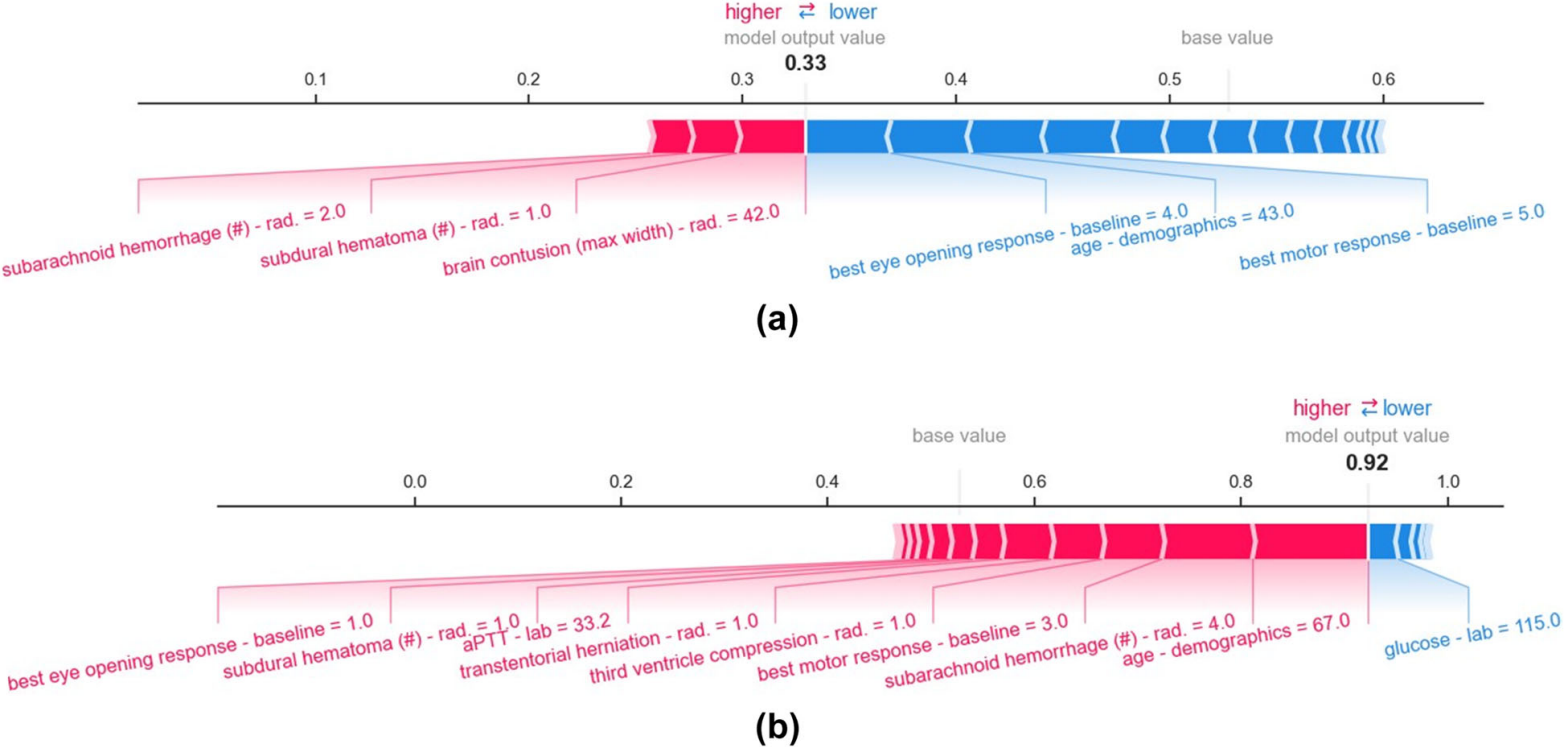
SHAP force plots corresponding to predicted risk scores for individuals. The base value corresponds to the average model output over the training set and is the proportion of the training samples belonging to the class GOSE 1–4. Red and blue arrows, respectively, depict the amount of positive and negative contribution of variables to the predicted risk score. The model output value corresponds to the predicted risk score. For example, the patient shown in plot **a** has a 33% probability of experiencing GOSE 1–4, while the patient shown in **b** has a 92% probability of experiencing GOSE 1–4. Variable types are denoted as *rad*: radiology report and *lab*: laboratory value.

The SHAP contribution values provide a detailed view into the risk factors leading to the probability risk score at both the population (Fig. 3 and Supplementary Fig. 4) and individual (Fig. 4) levels. At the population level, age and the number of brain regions with subarachnoid hemorrhage are by far the most impactful features in determining the elevated risk of poor outcome (Fig. 3). As can be observed in Fig. 3a, the contribution of a variable may vary across different patients even if the patients share the same value for that variable. For example, compression of the third ventricle can increase the risk of poor outcome from 3.97% to 6.60% depending on the combination of other risk factors.

At the individual level, each feature returns a contribution. The aggregate of all feature contributions yields the predicted risk score. For example, for patient shown in Fig. 4a, the presence of subarachnoid hemorrhage in two brain regions increases the predicted risk by 1.84%, while the eye opening response at the time of admission reduces the risk by 4.03%.

The most impactful features for the patient shown in Fig. 4a are age, eye opening response, motor response, subarachnoid hemorrhage, brain contusion, and subdural hematoma are different from the features that contribute to predicted risk score of the patient shown in Fig. 4b.

## Discussion

This was a secondary analysis of data from the ProTECT III data set, a large clinical trial of patients with moderate and severe TBI. An explainable, expert-guided machine learning framework was developed to automatically predict the long- term functional outcome of TBI patients as defined by GOSE. It is widely acknowledged that transparency and trustworthiness of machine learning models are important factors in real-world applicability, particularly in medical diagnostic and prognostic systems. The proposed framework seeks to move beyond the black box application of machine learning algorithms. SHAP values were used to estimate the contribution of each variable to the predicted risk scores at both the population and individual levels. Studying the contributions at the global level enables two rounds of variable selection to be performed, based on: (1) robustness of the contribution of a variable, and (2) clinical domain knowledge.

**Fig. 5:**
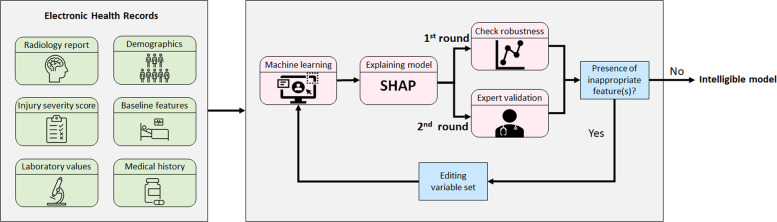
The proposed framework for developing an intelligible TBI prognostic model. After training an initial machine learning model, input features are selected based on statistical robustness and clinical validity. The machine learning model is retrained after each step of feature selection.

Among 62 candidate variables from EHR, 21 demonstrated robust global behavior where the global behavior was modeled using Kendall’s *τ* correlation coefficient. Of the 21 robust variables, 3 variables (active substance abuse, active gastrointestinal disease, and platelet count) showed counterintuitive effects on the predicted risk score. Based on the observed behaviors patients with active substance abuse and active gastrointestinal disease were determined to have a better chance of favorable outcome. The lower platelet count was found to be associated with favorable outcomes, which contradicts the clinical literature^32,33^. These three variables were excluded from the study as well, leaving 18 robust and clinically validated variables to be included in the final prognostic model.

Finally, an XGBoost classifier was trained to classify patients as having unfavorable (GOSE ≤ 4) or favorable (GOSE > 4) expected outcomes. The final model achieved an AUC, accuracy, and F1 score of 0.8085, 0.7488, and 0.7045, respectively, on the test set. Importantly, the results show that the performance of the model is not negatively affected by reducing the input variable set after imposing the statistical and domain knowledge constraints (Table 2). Tree-based models, including XGBoost, are prone to overfitting in the presence of many initial variables. This is evinced in Table 2, where the performance of XGBoost on the training set decreased after removing non-robust and counterintuitive variables.

In the final model, age and the total number of brain regions with subarachnoid hemorrhage were the most impactful features in predicting the risk score. These variables were followed by GCS motor score, intra-ventricular hemorrhage, GCS eye opening response, third ventricle compression, and subdural hematoma. Among laboratory values, hemoglobin, glucose, aPTT (activated Partial Thromboplastin Time), and INR (International Normalized Ratio) were determined to be appropriate predictors.

At the individual level, the model enabled the predicted risk score to be analyzed with respect to which risk factors contributed to a particular decision and to what extent. This tool enables end-users to judge the rationale behind the models’ decision making and act accordingly.

To our knowledge, no prior study has used explanatory methods such as SHAP values to select intelligible variables for black box machine learning classifiers. Using SHAP values for model explanation has become increasingly popular^35^. It is a powerful tool to peer inside black box models and understand how they arrive at a particular decision. Multiple recent studies used only SHAP values for variable selection^35–39^, however, only the variables with the greatest impact as defined by average absolute SHAP value were chosen. This is in contrast to this study, in which it was shown that variables with the greatest contribution are not necessarily robust (Fig. 1 and Supplementary Fig. 2a). For example, in the initial model, creatinine level was among the most impactful variables, while through the bootstrapping experiment it was shown to have non-robust behavior (Fig. 1). Moreover, the selected high impact features might not align with domain knowledge. For example, in Ogura et al.^36^, the total number of traumatic injuries was attributed to a lower risk of death. Thus, our study proposes a framework to “intelligibly” select variables using SHAP values, and highlights the importance of collaboration with domain experts.

GOSE at 6-months is the global gold standard functional outcome classification score in TBI prognostication studies. This score is commonly used in major clinical trials, such as ProTECT^31^ and RescueICP^40^. However, TBI patient functional outcome can be influenced by non-TBI-related post-injury adverse events. For example, in the test data set, there was a 40-year-old patient that was admitted to the hospital with a 23 mm unilateral subdural hematoma, with no sign of subarachnoid hemorrhage or increased intracranial pressure (e.g., third ventricle compression and transtentorial herniation) indicated in the radiology report. This patient’s best motor and eye opening responses were both 4 and within their respective upper quartiles. The patient was predicted to have a 27% chance of poor outcome; however, in reality, the patient died. Looking into post-admission information, the patient developed pneumonia after discharge from the hospital at day 86 post-injury, leading to the patient’s death. This is not the only case of non-TBI-related adverse events experienced post-injury. In the training set there was a 36-year-old subject that, except for 9 mm-wide intraparenchymal hematoma in one brain region, showed no other head abnormalities based on the radiology report. This patient was discharged to home at day 5 post-injury but died of a gun shot at day 87 post-injury. Although it is important, the information about non-TBI adverse events is not recorded for all patients, and even for the patients with such information, it is not mentioned in the data set whether functional GOSE outcome is derived by non-TBI adverse events or TBI alone. To avoid any subjective input, in particular in non-death cases, we did not consider post-injury clinical consolidated comorbidities as an exclusion criteria in this study. Though this limitation of GOSE introduces noise into the ground truth labels that can adversely affect machine learning performance, it is nonetheless the current best proxy for TBI outcomes. It should also be noted that the choice of thresholds, such as a GOSE > 4 being defined as a favorable outcome in this study, is somewhat arbitrary and lacks nuance. Ideally, equally validated but more detailed and objective means to measure TBI outcomes will become available for future studies.

It is also important to acknowledge that there can be “self-fulfilling prophecies” in clinical settings that can influence model performance in ways that are very challenging to mitigate. Self-fulfilling prophecies occur when a perceived poor prognostic factor is present, leading to early withdrawal of care, which then is seen as providing evidence that the prognostic factor is valid^3,41,42^_._

In addition to imperfect labels, the input variables are susceptible to bias or error. ProTECT III was conducted at 49 trauma centers in the United States^43^. Given this fact, there exists a potential level of noise or measurement error due to the subjectivity involved in radiology readings, the intrinsic differences in tools for measuring laboratory values, human error during data entry process, among other sources. These measurement errors in EHR can lead to potential loss of predictive power as well^44^. Given these aforementioned limitations - imperfect labeling, self-fulfilling prophecy, and measurement error - it is important to be cautious when applying models to individual patients.

Finally, the generalizability of the proposed model needs to be validated on an external data set and its utility determined prospectively in a clinical setting prior to its incorporation into standard practice. This was not undertaken in this study due to limited data availability. However, we believe that incorporating human domain knowledge into the model can potentially compensate for the lack of generalizability inherent in most black box machine learning methods. Involvement of clinicians in the model development process can help to eliminate suspect variables from the model whose presence may be due to confounding or statistical artifact, thereby increasing the likelihood that physicians will utilize the model.

In conclusion, a machine learning framework was proposed that enabled the creation of an explainable model for individualized-level prognostication of TBI functional outcomes. The proposed framework is transparent with respect to understanding how input variables result in the model’s decision at both the individual and patient population levels. Such a model can achieve high accuracy, avoid collinearity-induced biases, and ultimately accelerate adoption of machine learning models in clinical settings.

## Methods

The proposed framework for TBI outcome prediction is outlined in Fig. 5. First, a machine learning classifier is trained to predict the risk score for each patient (Machine Learning Module Section), with SHAP values being used to explain the model’s predictions (Explanation Module Section). Next, the global behavior of the SHAP values for each input variable is evaluated, with only those shown to be statistically robust selected for further consideration. These robust variables are then validated by a multidisciplinary team of clinical experts, with features with counterintuitive behavior investigated further and excluded to avoid potential sources of bias (Intelligible Variable Selection Module Section). Although excluding counterintuitive variables might negatively affect the overall accuracy, it is an essential step to develop a sensible and trustworthy algorithm that can be used operationally in a clinical setting.

**Fig. 6:**
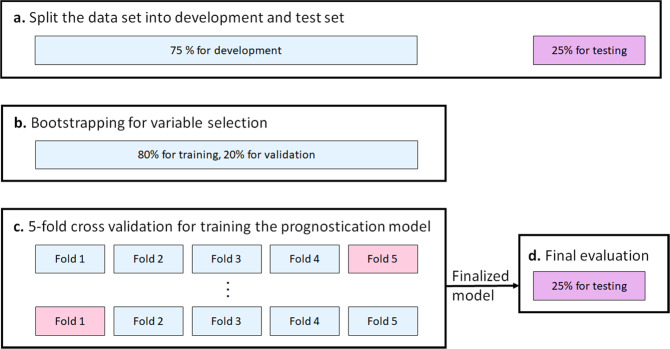
The experimental design. **a** At study onset, 25% of the data was set aside for final evaluation. The remaining 75% was used to develop the model, either in **b** the bootstrap step for variable selection, or **c** training the prognostic model using 5 fold cross-validation.

The experimental design is outlined in Fig. 6. To ensure that the final prognostic model is not affected by the test set, 25% of the whole data set will be randomly selected and set aside. This test data set will remain untouched until the prognostic model is trained and finalized.

### Data pre-processing

First, among all EHR variables available in the ProTECT III data set, those available at the time of admission were selected. Next, these selected variables were reviewed by an expert board-certified physician in neurology and neurocritical care, with all those deemed clinically relevant chosen as input variables. Information regarding race or ethnicity was excluded, as this information could reinforce an unwanted retrospective bias and/or discrimination rather than a direct cause of the predicted outcome^34,45^. Missing values were replaced by the average of available cases in the training set.

### Machine learning module

The XGBoost (eXtreme Gradient Boosting) algorithm was employed to classify each patient as experiencing either a favorable or unfavorable outcome at 6 months and to estimate its corresponding probability. XGBoost is a sequential tree growing algorithm with weighted samples. Compared to other boosting methods, XGBoost incorporates regularization parameters, making it relatively robust against noise and outliers while reducing over-fitting. The XGBoost package in Python was used to build this prognostic model^46^.

### Explanation module

SHAP (SHapley Additive exPlanations)^28,29^ and LIME (Local Interpretable Model-Agnostic Explanations)^47^ are two popular prediction explaining techniques^48^. In this study, the machine learning model’s outcome predictions were explained using SHAP as it leverages a theoretical foundation of cooperative game theory to unify multiple other feature explanation methods, including LIME^28,29^. Contrary to the SHAP method, LIME is calculated based on the assumption of local linearity of decision boundaries, which does not necessarily hold true^28,49^.

In game theory, the Shapley value fairly distributes both gains and costs between multiple players with different skill sets in a coalition. Inspired by this concept, in the machine learning context, SHAP values can fairly distribute a predicted probability among input features. This distribution can be either positive or negative. The positive contribution of a variable indicates that it increases the prediction probability, while a negative contribution denotes a reduction in that probability. Accordingly, SHAP values enable model interpretation at both the individual patient and population levels.

At the patient level, SHAP estimates the contribution of each variable to the predicted outcome. It provides a sense of which variables are contributing to the predicted outcome and to what extent. In the aggregate, the distribution of SHAP values over the whole data set reveals the global behavior of the trained model and input features. In this study, SHAP values were calculated using the Tree SHAP algorithm available in the SHAP Python package developed by Lundburg and Lee^28,29^. Tree SHAP is a computationally efficient algorithm to estimate SHAP values for tree ensemble models^29^.

### Intelligible variable selection module

**Step 1 - Statistical analysis to identify variables with robust contribution behaviors**. While the global behavior of SHAP values for some variables is robust regardless of the sampled training set, there are variables for which their global contribution distribution vary based on the selected training sample set. For example, the global behavior of creatinine contribution is highly sensitive to the training sample set. As shown in Fig. 1 depending on the randomly selected training sample set, the marginal effect of creatinine contribution significantly fluctuates. To identify and exclude variables with non-robust and unreliable SHAP contribution behavior, a bootstrap-based procedure was employed. 1000 bootstrap samples were drawn with replacement from the training set. For each bootstrap sample a separate XGBoost model was trained. Next, the SHAP contribution behavior was estimated for each variable using Kendall’s *τ* correlation coefficient. Kendall’s *τ* is a summary statistic that in this usage assesses the strength and direction of the association between a variable and its SHAP contribution. For each variable, if its correlation coefficient, either positive or negative, is marginally significant with *p*-value < 0.1, variable is selected to be included in the remainder of the process. The choice of the *p-*value is arbitrary. In this study, since there is a subsequent variable selection step that examines variable behavior in detail from a clinical perspective, only variables whose behavior was strongly non-significant (*p*-value > 0.1) were excluded during the statistical inference process.

**Step 2 - Clinical expert validation of input variables**. Once the robust features are selected, the XGBoost classifier is again trained and its predictions are explained using SHAP values. Next, human experts investigated the model explanation in order to complement the machine learning approach. An interdisciplinary team of an expert board-certified physician in neurology and neurocritical care, data scientists, and engineers studied each input variable’s contribution to the final outcome prediction. SHAP values of the whole population are used to investigate each variable’s marginal effect on the predicted probability. If a variable showed a counterintuitive behavior it was further studied for potential sources of biases. If no logical explanation could be derived, the variable was excluded from the study. Finally, using a robust and medically justified subset of the variables in the XGBoost model, the TBI prognostic model was developed.

### Reporting summary

Further information on research design is available in the Nature Research Reporting Summary linked to this article.

## Supplementary information

Supplementary Information

Reporting Summary

## Data Availability

The data sets generated during and/or analyzed during the current study were shared with the University of Michigan through a non-disclosure agreement (NDA). We are not able to share the data under the terms of this NDA. Qualified researchers may contact the Progesterone for Traumatic Brain Injury Experimental Clinical Treatment (ProTECT) III Trial’s Principal Investigator for potential access to the data set.

## Code availability

The executable code developed in this work is available from the corresponding author upon the University of Michigan’s Office of Technology Transfer approval.
