# Supplementary material for: A hierarchical expert-guided machine learning framework for clinical decision support systems: an application to traumatic brain injury prognostication
Source: NPJ Digit Med. 2021 May 7;4:78. doi: 10.1038/s41746-021-00445-0 (PMC8105342; doi:10.1038/s41746-021-00445-0)
Supplement: Supplementary file 1 — Supplementary Information [file 41746_2021_445_MOESM1_ESM.pdf]

Supplementary Information: A Hierarchical Expert-Guided Machine Learning Framework for Clinical Decision Support Systems: An Application to Traumatic Brain Injury Prognostication

Supplementary Methods

Machine Learning Algorithm Selection

To select the machine learning algorithm to be used in this study, we trained five different algorithms including XGBoost, deep learning, logistic regression, and support vector machine using the initial 62 candidate features. For deep learning, we used feed forward neural network with 4 hidden layers. The model that performed the best on the validation set is then chosen for the remainder of the process. Supplementary Table 2 compares the performance of the candidate machine learning algorithms. Although the performance on the validation is used to select the model, for more information, we included the performance on the training and test sets in Supplementary Table 2 as well. XGBoost model outperformed the other methods and was chosen in our TBI prognostication study.

Hyperparameter Tuning

The hyperparameters were optimized for all models using grid search over a specified subset of the hyperparameter space. For each model, the combination of the hyperparameters that yielded the maximum F1 score was selected. This combination was calculated based on the validation set performance.

Supplementary Results

Supplementary Table 4 compares the predictive performance of multiple classifiers, including XGBoost, deep learning, logistic regression, and support vector machine, using the 18 selected variables after excluding non-robust and counterintuitive variables. It can be concluded that after feature selection, the predictive performance of logistic regression outperforms the XGBoost model. However, the logistic regression model's log-odds coefficient for the variable INR is negative (log-odds = -0.04), contrary to domain knowledge. A higher INR value leads to a greater risk of bleeding, and thus, a worse outcome, while the logistic regression coefficient contradicts it. This result might suggest that it is preferable for each classifier to use a feature selection method based on its underlying mathematical assumptions.

Supplementary Tables

Supplementary Table 1. List of candidate and selected variables and their definitions. [a] Subarachnoid hemorrhage refers to bleeding into the subarachnoid space between the brain and the surrounding membrane. Brain regions include suprasellar, basal cisterns, right and left Sylvian fissure, right and left interhemispheric, right and left lobar-frontal, right and left lobar-parietal, right and left lobar-occipital, right and left lobar temporal. [b] Intraparenchymal hemorrhage refers to bleeding within the brain parenchyma. Brain regions include midbrain/pons, right and left frontal, right and left temporal, right and left parietal, right and left occipital, right and left basal ganglia, right and left posterior fossa. [c] Brain contusions refer to the bruises of the brain tissue. Brain regions include midbrain/pons, right and left frontal, right and left temporal, right and left parietal, right and left occipital, right and left basal ganglia, right and left posterior fossa. [d] Diffuse Axonal Injury (DAI) corresponds to shearing of the brain's axons due to brain shifts or rotations after an injury. Brain regions include right and left frontal, right and left parietal, right and left basal ganglia, brainstem, corpus callosum, right and left centrum semiovale. [e] Brain regions include midbrain/pons, right and left frontal, right and left temporal, right and left parietal, right and left occipital, right and left basal ganglia.

**Name**	**Definition (unit)**	**Median (min–max)**	**No: Yes or None: One: Two**	**Selected in Final Model**
Demographics				
Age		35 (17-94)		Yes
Sex: female			607 (73.04%): 224 (26.96%)	
Baseline features				
Best motor response	As defined by[1]	4 (1-6)		Yes
Best eye opening response	As defined by[1]	1 (1-4)		Yes
Best verbal response	As defined by[1]	1 (1-5)		Yes
Pupil response	None, one, or both eyes		35 (4.2%): 125 (15.0%): 671 (80.7%)	
Radiology report				
Epidural hematoma (#)	Zero if none, one if unilateral, and two if bilateral epidural hematoma		710 (85.44%): 110(13.24%): 11(1.32%)	
Epidural hematoma (max width)	(mm)	0 (0-102)		
Subdural hematoma (#)	Zero if none, one if unilateral, and two if bilateral subdural hematoma		425 (51.1%): 330 (39.7%): 76 (9.1%)	Yes
Subdural hematoma (max width)	(mm)	0 (0-125)		Yes
Subarachnoid hemorrhage (#)	Number of brain regions with subarachnoid hemorrhage[a]	1 (0-14)		Yes
Intra-ventricular hemorrhage	Zero if none, one if minimal layering, and two if clot intra-ventricular hemorrhage		642 (77.3%): 119 (14.3%): 70 (8.4%)	Yes
Intraparenchymal hematoma (#)	Number of brain regions with intraparenchymal hemorrhage[b]	0 (0–4)		
Intraparenchymal hematoma (max width)	(mm)	0 (0-67)		Yes
Evidence of surgical evacuation	Evidence of surgical evacuation of intraparenchymal hematoma		827 (99.52%): 4 (%0.48)	

Supplementary Table 1 continued from previous page

Name	Definition (unit)	Median (min–max)	No: Yes or None: One: Two	Selected in Final Model
Brain contusion (#)	Number of brain regions with brain contusion [c]	0 (0-5)		Yes
Brain contusion (max width)	(mm)	0 (0-93)		Yes
DAI finding (#)	Number of brain regions with diffuse axonal injury [d]	0 (0-6)		Yes
DAI finding (max width)	Maximum width of diffuse axonal injury (mm)	0 (0–22)		
Generalized edema severity	Zero if none, one of mild, and two if moderate edema		654 (78.70%): 52 (6.26%): 125 (15.04%)	
Focal swelling (#)	Number of brain regions with focal swelling [e]	0 (0-3)		
Midline shift	Shift of over 5 mm		705 (84.84%): 126 (15.16%)	
Sulcal obliteration	Zero if none, one if unilateral, and two if bilateral sulcal obliteration		643 (77.38%): 77 (9.27%): 111 (13.36%)	
Lateral ventricle compression			835 (76.41%): 159 (19.13%)	
Third ventricle compression			653 (78.6%):: 178 (21.4%)	Yes
Transtentorial herniation			700 (84.2%): 131 (15.8%)	Yes
Uncal herniation			714 (85.92%): 117 (14.08%)	
Tonsillar herniation			767 (92.30%): 64 (7.70%)	
Upward herniation			819 (98.56%): 12 (1.44%)	
Depressed skull fracture			773 (93.02%): 58 (6.98%)	
Basilar skull fracture			646 (77.74%): 185 (22.26%)	
Abbreviated Injury Scores				
Neck		4 (0-6)		
Face		0 (0–4)		
Chest		1 (0-5)		
Abdomen		0 (0–5)		
Extremity		1 (0–5)		
External skin		1 (0–4)		
Laboratory values				
Glucose	(mg/dL)	143 (68-554)		Yes

Supplementary Table 1 continued from previous page

Name	Definition (unit)	Median (min–max)	No: Yes or None: One: Two	Selected in Final Model
Creatinine	(mg/dL)	1.0 (0.3-4.2)		
Potassium	(mmol/L)	3.7 (1.5-6.5)		
Sodium	(mmol/L)	140 (125-157)		
Chloride	(mmol/L)	105 (88-130)		
Bicarbonate	(mmol/L)	23 (8-34)		
Hgb	Hemoglobin (g/dL)	13.8 (2.0-18.7)		Yes
WBC	White blood cell count ($\times 10^9$/L)	13.6 (3.2-41.4)		
Platelets	Platelet count ($\times 10^3$/mm^3)	237 (36-700)		
aPTT	Activated partial thromboplastin time (sec)	26 (12-73)		Yes
INR	International Normalized Ratio	1.1 (0.8-12.0)		Yes
Medical history				
Active neurological disease	Includes prior TBI hospitalization or medical evaluation, CVA, seizure, paralysis/neurological weakness, headache, sleep disorder, and other unknown		804 (96.75%): 27 (3.25%)	
Inactive neurological disease	Prior neurological disease		735 (88.45%): 96 (11.55%)	
Active cardiovascular disease	Includes heart disease, hypertension, arrhythmias, and other unknown		692 (83.3%): 139 (16.7%)	
Inactive cardiovascular disease	Prior cardiovascular disease		787 (94.71%): 44 (5.29%)	
Active pulmonary disease	Includes COPD or asthma, and other unknown		779 (93.73%): 52 (6.26%)	
Inactive pulmonary disease	Prior pulmonary disease		793 (95.43%): 38 (0.4.57%)	
Active metabolic disease	Includes diabetes mellitus, pituitary disease, and other unknown		760 (91.46%): 71 (8.54%)	
Inactive metabolic disease	Prior metabolic disease		827 (99.52%): 4 (0.48%)	
Active gastrointestinal disease	Includes liver disease, hepatitis, and other unknown		768 (92.42%): 63 (7.58%)	
Inactive gastrointestinal disease	Prior gastrointestinal disease		800 (96.27%): 31 (3.73%)	
Active psychiatric disease	Includes depression/ suicidal gestures, schizophrenia, anxiety, and other unknown		699 (84.12%): 132 (15.88%)	

Supplementary Table 1 continued from previous page

**Name**	**Definition (unit)**	**Median (min–max)**	**No: Yes or None: One: Two**	**Selected in Final Model**
Inactive psychiatric disease	Prior psychiatric disease		802 (96.51%): 29 (3.49%)	
Active substance abuse	Alcohol and non-prescribed drug abuse		592 (71.24%): 239 (28.76%)	
Inactive substance abuse	Prior substance abuse		782 (94.10%) 49 (5.90%)	

Supplementary Table 2. The Kendall's τ correlation coefficients and the corresponding p-values of variables that demonstrated robust or non-robust SHAP global behavior.

**Robust Variables**		
**Variable name**	**Median**	***p*-value**
Age	0.74	<0.001
Best motor response	-0.75	<0.001
Subarachnoid hemorrhage (#)	0.79	<0.001
Intra-ventricular hemorrhage	0.60	<0.001
Best eye opening response	-0.66	0.003
Hgb	-0.76	0.003
Transtentorial herniation	0.52	0.006
Best verbal response	-0.76	0.007
Third ventricle compression	0.59	0.012
Subdural hematoma (max width)	0.64	0.018
Brain contusion (max width)	0.56	0.018
Subdural hematoma (#)	0.68	0.019
DAI finding (#)	0.45	0.025
Platelets	0.67	0.039
Glucose	0.52	0.054
Intraparenchymal hematoma (max width)	0.28	0.066
Brain contusion (#)	0.50	0.084
Active gastrointestinal disease	-0.40	0.086
aPTT	0.59	0.089
Active substance abuse	-0.65	0.088
INR	0.62	0.087
**Non-robust Variables**		
**Variable name**	**Median**	***p*-value**
Pupil response	-0.55	0.105
Active cardiovascular disease	0.53	0.120
DAI finding (max width)	0.41	0.130
Basilar skull fracture	0.61	0.162
Intraparenchymal hematoma (#)	0.32	0.169
Abdomen injury severity score	-0.57	0.170
Creatinine	-0.45	0.182
Potassium	0.48	0.184
Chest injury severity score	0.59	0.188
Lateral ventricle compression	0.55	0.210
Extremity injury severity score	0.59	0.232
Bicarbonate	0.28	0.248
Uncal herniation	0.50	0.251
Generalized edema severity	0.57	0.271
External skin injury severity score	-0.41	0.293
Sulcal obliteration	0.57	0.302
WBC	-0.32	0.304
Face injury severity score	-0.47	0.323
Inactive gastrointestinal disease	0.27	0.330
Inactive neurological disease	-0.47	0.358
Midline shift	0.52	0.374
Neck injury severity score	0.26	0.382
Depressed skull fracture	-0.39	0.390
Chloride	0.17	>0.400
Active pulmonary disease	-0.36	>0.400
Gender	-0.62	>0.400

Supplementary Table 2 continued from previous page

**Variable name**	**Median**	***p*-value**
Sodium	-0.06	>0.400
Focal edema (#)	-0.32	>0.400
Tonsillar herniation	0.40	>0.400
Active psychiatric disease	-0.50	>0.400
Epidural hematoma (max width)	-0.04	>0.400
Inactive substance abuse	-0.33	>0.400
Inactive cardiovascular disease	0.30	>0.400
Epidural hematoma (#)	0.00	>0.400
Inactive psychiatric disease	-0.27	>0.400
Active metabolic disease	0.09	>0.400
Inactive pulmonary disease	-0.29	>0.400
Active neurological disease	-0.07	>0.400
Evidence of surgical evacuation	-0.11	>0.400
Upward herniation	0.20	>0.400
Inactive metabolic disease	0.14	>0.400

Supplementary Table 3. Comparing the performance of multiple machine learning algorithms in predicting GOSE ≤ 4 using initial candidate variables. Standard deviation (SD) is calculated over 5 cross-validation folds.

**Training Set**				
**Method**	**XGBoost**	**Deep learning**	**Logistic regression**	**Support vector machine**
AUC (SD)	0.9372 (0.0236)	0.9403 (0.0482)	0.8681 (0.0043)	0.8193 (0.0135)
Accuracy (SD)	0.8522 (0.0327)	0.8702 (0.0703)	0.7868 (0.0136)	0.7468 (0.0240)
F1 (SD)	0.8281 (0.0360)	0.8467 (0.0747)	0.7527 (0.0106)	0.6613 (0.0409)
Sensitivity (SD)	0.8477 (0.0305)	0.8324 (0.0441)	0.7749 (0.0187)	0.5939 (0.0545)
Specificity (SD)	0.8554 (0.0440)	0.8974 (0.1012)	0.7954 (0.0319)	0.8568 (0.0116)
Precision (SD)	0.8106 (0.0529)	0.8677 (0.1194)	0.7329 (0.0273)	0.7479 (0.0226)
**Validation Set**				
**Method**	**XGBoost**	**Deep learning**	**Logistic regression**	**Support vector machine**
AUC (SD)	0.7822 (0.0126)	0.7607 (0.0269)	0.7652 (0.0304)	0.7838 (0.0206)
Accuracy (SD)	0.7500 (0.0169)	0.7051 (0.0398)	0.7164 (0.0225)	0.7500 (0.0334)
F1 (SD)	0.7129 (0.0190)	0.6540 (0.0364)	0.6735 (0.0377)	0.6652 (0.0492)
Sensitivity (SD)	0.7434 (0.0489)	0.6669 (0.0762)	0.7055 (0.0828)	0.5978 (0.0695)
Specificity (SD)	0.7549 (0.0456)	0.7330 (0.0963)	0.7248 (0.0542)	0.8596 (0.0479)
Precision (SD)	0.6880 (0.0330)	0.6519 (0.0653)	0.6505 (0.0303)	0.7595 (0.0602)
**Test Set**				
**Method**	**XGBoost**	**Deep learning**	**Logistic regression**	**Support vector machine**
AUC	0.8094	0.7790	0.8033	0.7695
Accuracy	0.7536	0.7290	0.7391	0.7005
F1	0.7052	0.6500	0.6747	0.5811
Sensitivity	0.7011	0.5977	0.6437	0.4943
Specificity	0.7917	0.8250	0.8083	0.8500
Precision	0.7093	0.7123	0.7089	0.7049

Supplementary Table 4. Comparing the performance of multiple machine learning algorithms in predicting GOSE ≤ 4 using the 18 selected variables after excluding non-robust and counterintuitive variables. Standard deviation (SD) is calculated over 5 cross-validation folds.

**Training Set**				
**Method**	**XGBoost**	**Deep learning**	**Logistic regression**	**Support vector machine**
AUC (SD)	0.8912 (0.0252)	0.9213 (0.0398)	0.8681 (0.0043)	0.8110 (0.0056)
Accuracy (SD)	0.8053 (0.0285)	0.8393 (0.0581)	0.7868 (0.0136)	0.7300 (0.0104)
F1 (SD)	0.7740 (0.0375)	0.7999 (0.0724)	0.7527 (0.0106)	0.6429 (0.0214)
Sensitivity (SD)	0.8018 (0.0637)	0.7681 (0.0801)	0.7749 (0.0187)	0.5824 (0.0323)
Specificity (SD)	0.8078 (0.0238)	0.8905 (0.0680)	0.7954 (0.0319)	0.8361 (0.0073)
Precision (SD)	0.7500 (0.0252)	0.8394 (0.0850)	0.7329 (0.0273)	0.7185 (0.0062)
**Validation Set**				
**Method**	**XGBoost**	**Deep learning**	**Logistic regression**	**Support vector machine**
AUC (SD)	0.7836 (0.0189)	0.7731 (0.0334)	0.8042 (0.0207)	0.7890 (0.0187)
Accuracy (SD)	0.7451 (0.0255)	0.7275 (0.0395)	0.7452 (0.0062)	0.7516 (0.0170)
F1 (SD)	0.7076 (0.0315)	0.6806 (0.0410)	0.7219 (0.0146)	0.6724 (0.0404)
Sensitivity (SD)	0.7393 (0.0570)	0.6936 (0.0600)	0.7932 (0.0532)	0.6169 (0.0785)
Specificity (SD)	0.7494 (0.0443)	0.7522 (0.0737)	0.7108 (0.0360)	0.8487 (0.0429)
Precision (SD)	0.6813 (0.0329)	0.6740 (0.0632)	0.6647 (0.0145)	0.7516 (0.0442)
**Test Set**				
**Method**	**XGBoost**	**Deep learning**	**Logistic regression**	**Support vector machine**
AUC	0.8085	0.7730	0.8201	0.8045
Accuracy	0.7488	0.7440	0.7488	0.7295
F1	0.7045	0.6748	0.7143	0.6216
Sensitivity	0.7126	0.6322	0.7471	0.5287
Specificity	0.7750	0.8250	0.7500	0.8750
Precision	0.6966	0.7239	0.6842	0.7541

Supplementary Figures

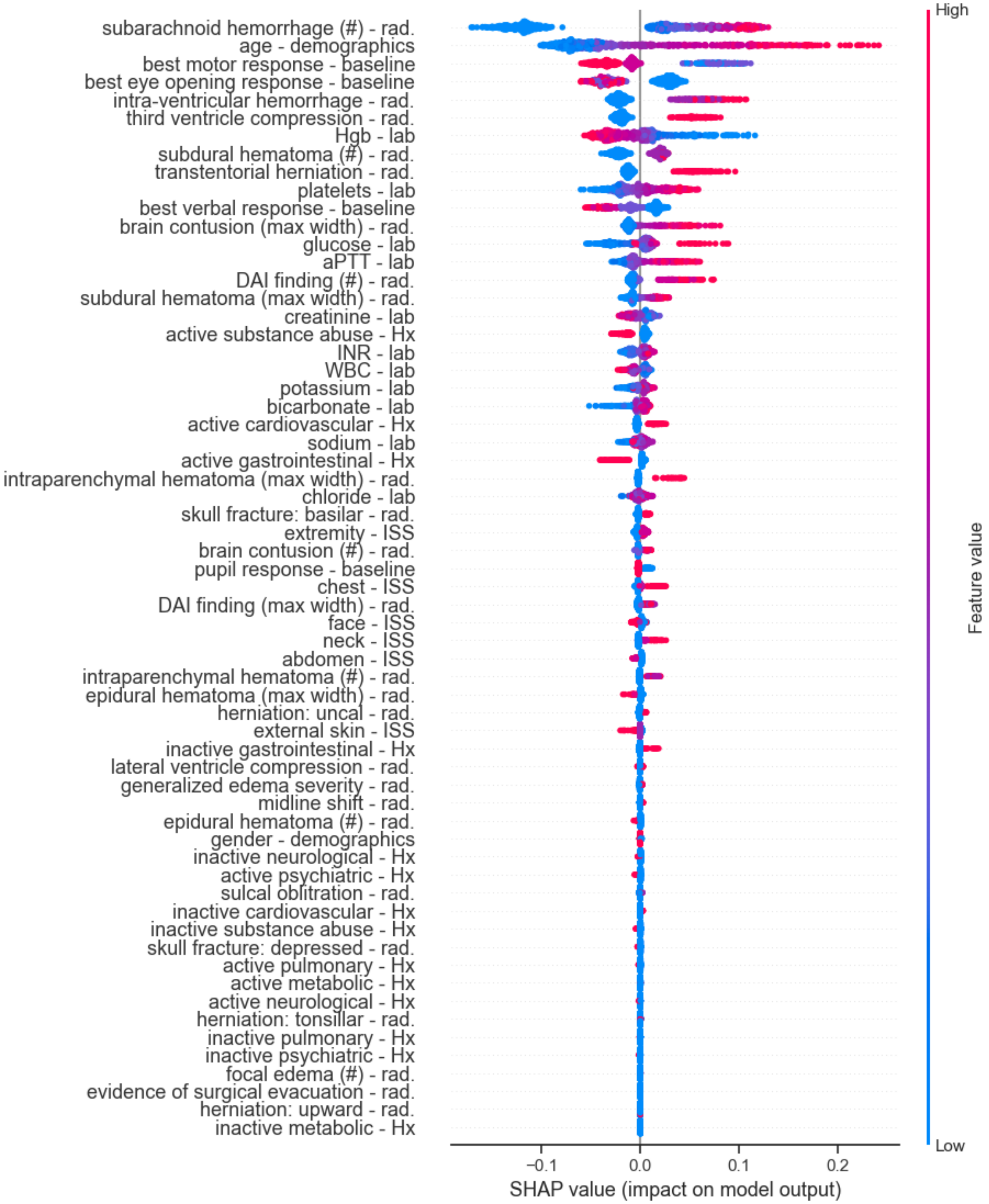

(a)

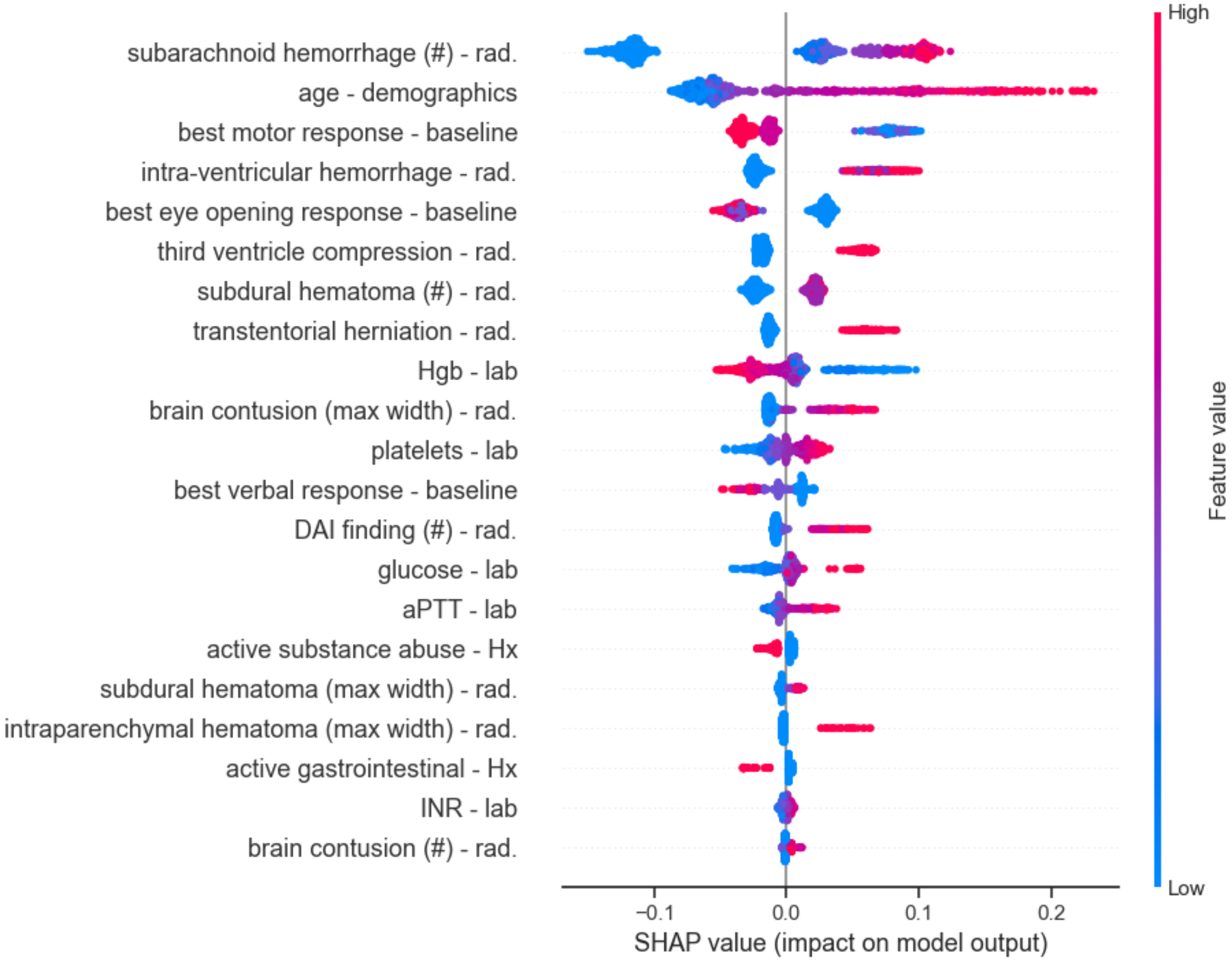

(b)

Supplementary Figure 1. Summary of SHAP contribution in the initial and intermediate model. (a) shows the summary of contributions in a model trained using the 62 candidate variables, (b) shows the summary of contributions in a model trained using the selected 21 robust variables. Variable types are denoted as follows - *rad*: radiology report, *lab*: laboratory value, *Hx*: medical history, and *ISS*: injury severity score.

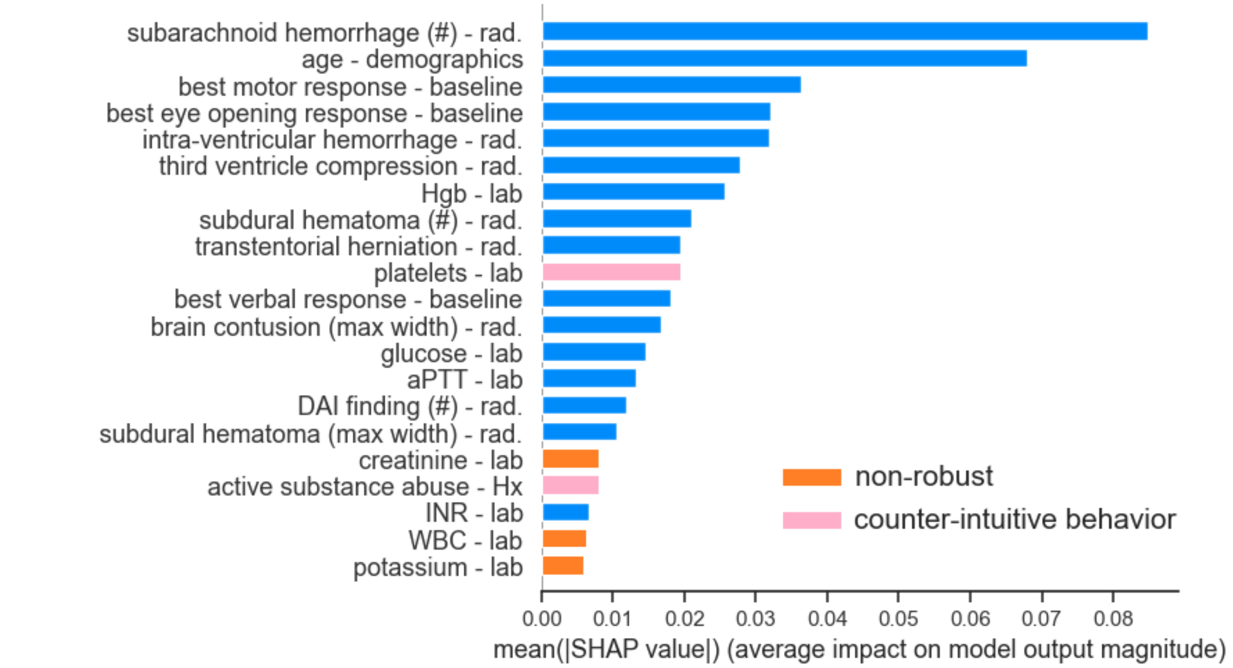

(a)

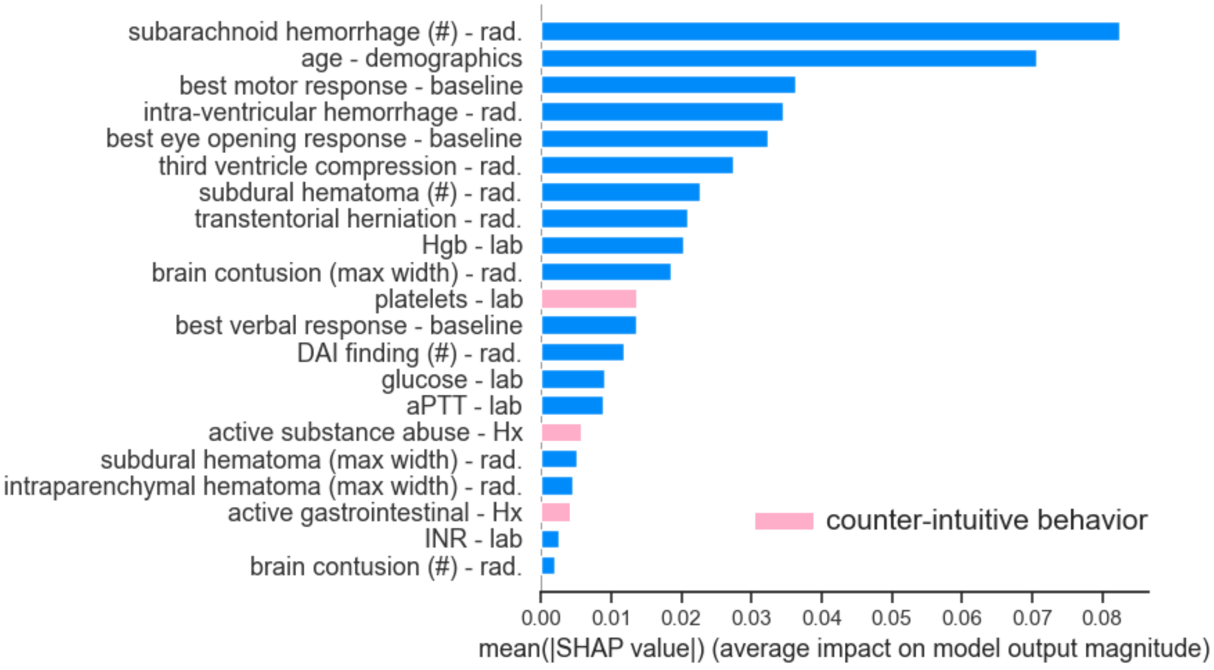

(b)

Supplementary Figure 2. Variables are shown in order of their average impact on the predicted risk, where impact is defined as the average absolute SHAP value. (a) corresponds to the initial model with 62 candidate features. Only 21 variables with the highest impact are shown in the plot. (b) corresponds to the model with 21 robust variables. Variable types are denoted as follows - *rad*: radiology report, *lab*: laboratory value, and *Hx*: medical history.

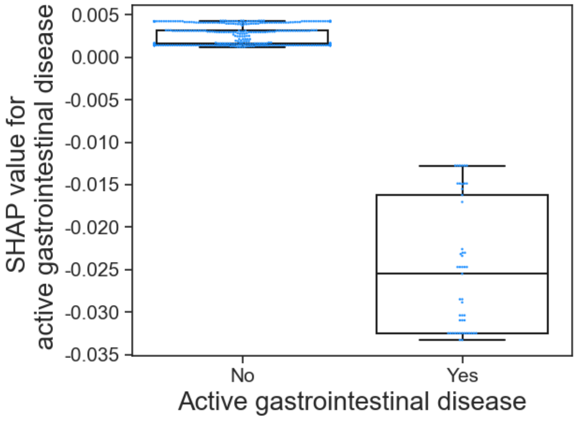

(a)

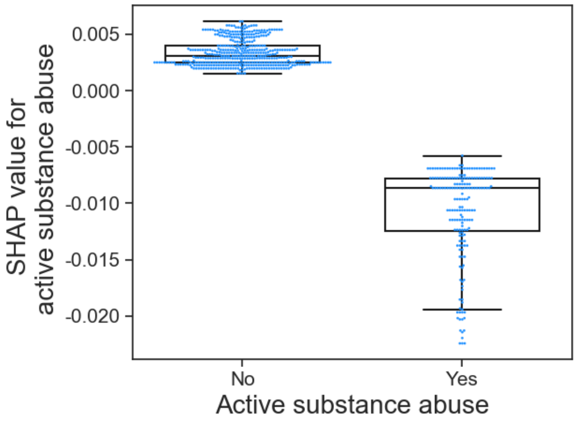

(b)

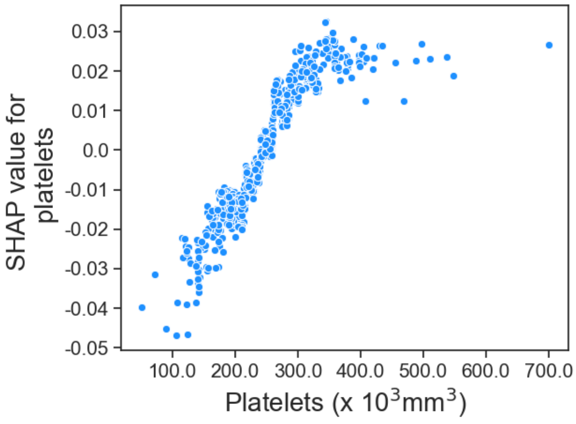

(c)

Supplementary Figure 3. SHAP contribution for variable with robust counterintuitive behavior. (a) shows the contribution of presence of active gastrointestinal disease contribution, (b) shows the contribution of active substance abuse, while (c) shows the contribution of platelet count.

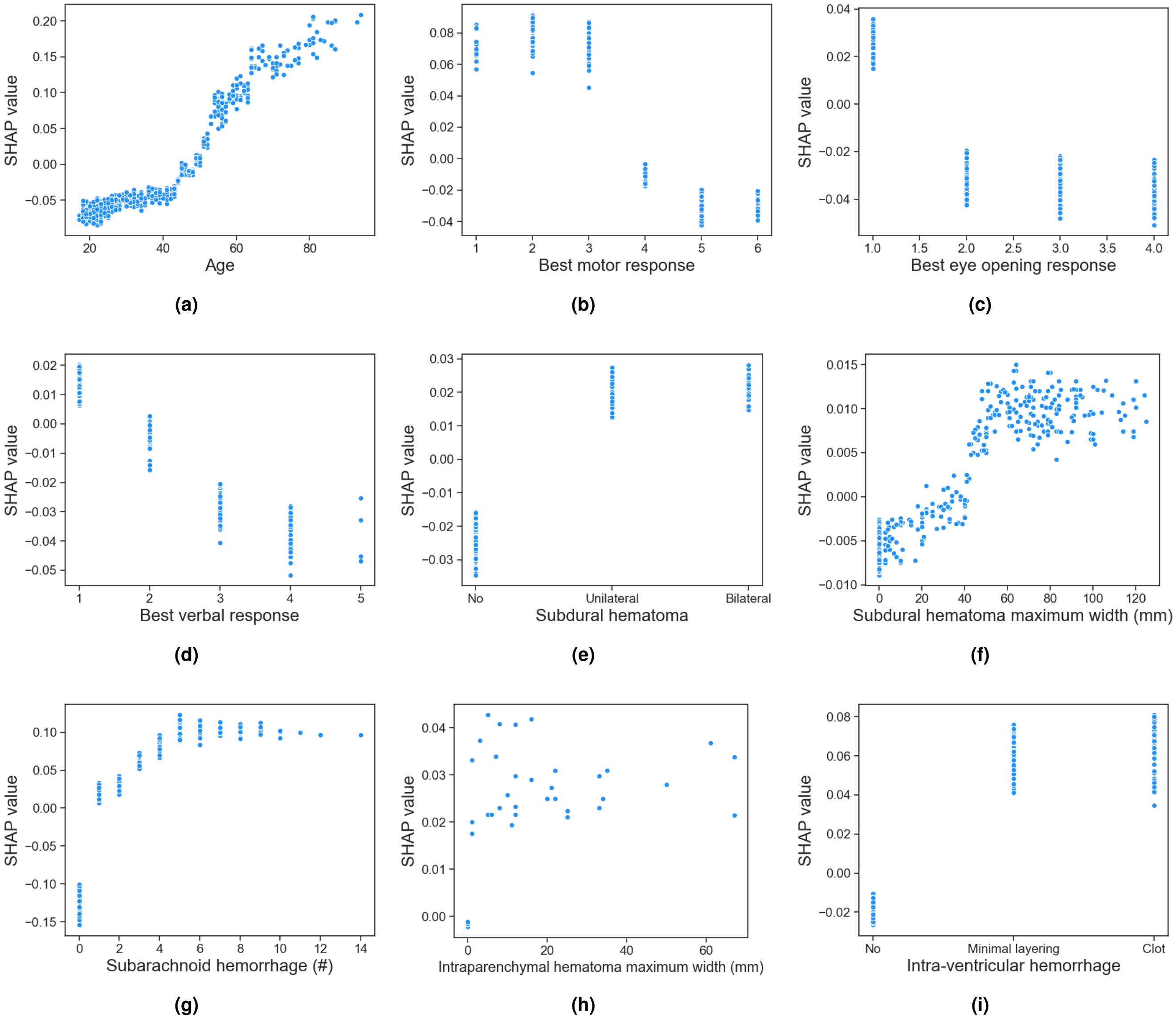
SHAP value
Age
(a)
Best motor response
(b)
Best eye opening response
(c)
Best verbal response
(d)
No
Unilateral
Bilateral
Subdural hematoma
(e)
Subdural hematoma maximum width (mm)
(f)
Subarachnoid hemorrhage (#)
(g)
Intraparenchymal hematoma maximum width (mm)
(h)
No
Minimal layering
Clot
Intra-ventricular hemorrhage
(i)

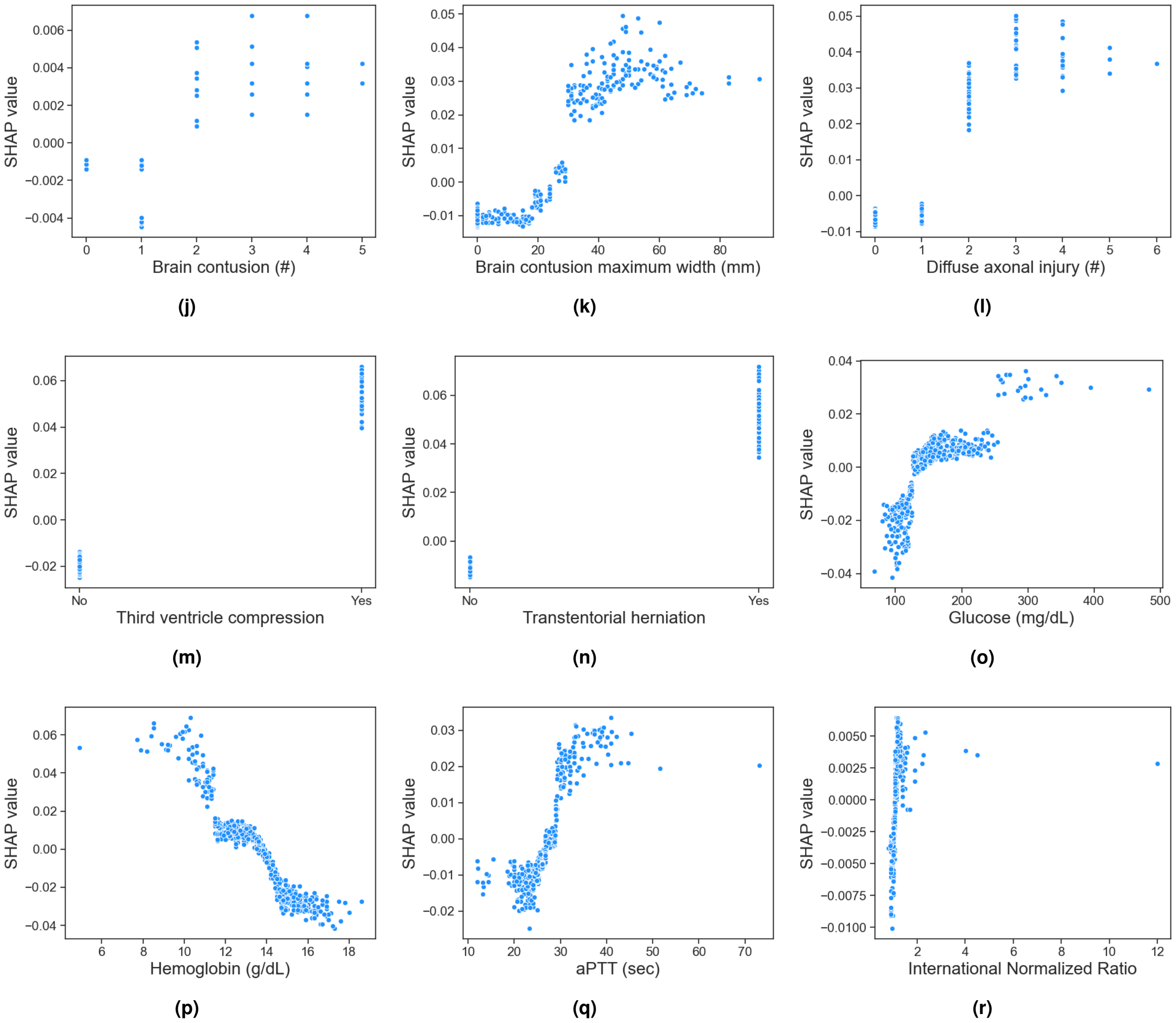

Supplementary Figure 4. Detailed contribution of the 18 selected features.

References

1. Teasdale, G. & Jennett, B. Assessment and prognosis of coma after head injury. *Acta neurochirurgica* **34**, 45–55 (1976).
