# Supplementary material for: A hierarchical expert-guided machine learning framework for clinical decision support systems: an application to traumatic brain injury prognostication
Source: NPJ Digit Med. 2021 May 7;4:78. doi: 10.1038/s41746-021-00445-0 (PMC8105342; doi:10.1038/s41746-021-00445-0)
Supplement: Supplementary file 2 — Reporting Summary [file 41746_2021_445_MOESM2_ESM.pdf]

nature research

Corresponding author(s): Negar Farzaneh

Last updated by author(s): 2021/03/22

Reporting Summary

Nature Research wishes to improve the reproducibility of the work that we publish. This form provides structure for consistency and transparency in reporting. For further information on Nature Research policies, see our Editorial Policies and the Editorial Policy Checklist.

Statistics

For all statistical analyses, confirm that the following items are present in the figure legend, table legend, main text, or Methods section.

n/a	Confirmed	
☐	☒	The exact sample size (n) for each experimental group/condition, given as a discrete number and unit of measurement
☐	☒	A statement on whether measurements were taken from distinct samples or whether the same sample was measured repeatedly
☒	☐	The statistical test(s) used AND whether they are one- or two-sided<br>*Only common tests should be described solely by name; describe more complex techniques in the Methods section.*
☐	☒	A description of all covariates tested
☒	☐	A description of any assumptions or corrections, such as tests of normality and adjustment for multiple comparisons
☐	☒	A full description of the statistical parameters including central tendency (e.g. means) or other basic estimates (e.g. regression coefficient) AND variation (e.g. standard deviation) or associated estimates of uncertainty (e.g. confidence intervals)
☒	☐	For null hypothesis testing, the test statistic (e.g. F, t, r) with confidence intervals, effect sizes, degrees of freedom and P value noted<br>*Give P values as exact values whenever suitable.*
☒	☐	For Bayesian analysis, information on the choice of priors and Markov chain Monte Carlo settings
☐	☒	For hierarchical and complex designs, identification of the appropriate level for tests and full reporting of outcomes
☒	☐	Estimates of effect sizes (e.g. Cohen's d, Pearson's r), indicating how they were calculated

Our web collection on statistics for biologists contains articles on many of the points above.

Software and code

Policy information about availability of computer code

Data collection	NA
Data analysis	We used MATLAB 2019 to read and prepare features from the electronic health records spreadsheet, and Python to perform all the remaining analysis.

For manuscripts utilizing custom algorithms or software that are central to the research but not yet described in published literature, software must be made available to editors and reviewers. We strongly encourage code deposition in a community repository (e.g. GitHub). See the Nature Research guidelines for submitting code & software for further information.

Data

Policy information about availability of data

All manuscripts must include a data availability statement. This statement should provide the following information, where applicable:

- Accession codes, unique identifiers, or web links for publicly available datasets
- A list of figures that have associated raw data
- A description of any restrictions on data availability

The data sets generated during and/or analyzed during the current study were shared with the University of Michigan through a non-disclosure agreement (NDA). We are not able to share the data under the terms of this NDA.

Field-specific reporting

Please select the one below that is the best fit for your research. If you are not sure, read the appropriate sections before making your selection.

☒ Life sciences ☐ Behavioural & social sciences ☐ Ecological, evolutionary & environmental sciences

For a reference copy of the document with all sections, see nature.com/documents/nr-reporting-summary-flat.pdf

Life sciences study design

All studies must disclose on these points even when the disclosure is negative.

Sample size	This is a secondary analysis of the Progesterone for the Treatment of Traumatic Brain Injury III (ProTECT) data set thatincludes adults who experienced a moderate to severe brain injury caused by blunt trauma. The data set includes electronic electronic data for 882 patient. Among the 882 patients, 831 met the inclusion criteria and were followed-up for functional outcome.
Data exclusions	Patients were excluded from ProTECT III if they had an initial Glasgow Comma Scale (GCS) of3, bilateral dilated unresponsive pupils, or were otherwise determined to have non-survivable injuries.
Replication	NA
Randomization	Data set randomly was split in the training (75%) and test (25%) set. We ensured that the proportion of positive to negative samples remains the same in each set by splitting in a stratified fashion using the class labels.
Blinding	Investigators were not blinded to the group allocations during the analysis. This was the secondary analysis of an existing data set and all labels were available at the time of this study. However, to ensure that the final prognostic model is not affected by the test set, 25% of the whole data set will be randomly selected and set aside. This test data set will remain untouched until the prognostic model is trained and finalized.

Reporting for specific materials, systems and methods

We require information from authors about some types of materials, experimental systems and methods used in many studies. Here, indicate whether each material, system or method listed is relevant to your study. If you are not sure if a list item applies to your research, read the appropriate section before selecting a response.

Materials & experimental systems

n/a	Involved in the study	
☒	☐	Antibodies
☒	☐	Eukaryotic cell lines
☒	☐	Palaeontology and archaeology
☒	☐	Animals and other organisms
☒	☐	Human research participants
☐	☒	Clinical data
☒	☐	Dual use research of concern

Methods

n/a	Involved in the study	
☒	☐	ChIP-seq
☒	☐	Flow cytometry
☒	☐	MRI-based neuroimaging

Clinical data

Policy information about clinical studies

All manuscripts should comply with the ICMJE guidelines for publication of clinical research and a completed CONSORT checklist must be included with all submissions.

Clinical trial registration	ClinicalTrials.gov identifier NCT00822900
Study protocol	Study protocol can be find in the following manuscript: Wright, D. W.et al.Very early administration of progesterone for acute traumatic brain injury.New Engl. J. Medicine371,2457–2466 (2014)
Data collection	The PROTECT III trial was conducted at 49 trauma centers in the United States. Data was collected between April 5, 2010, and October 30, 2013.
Outcomes	In our study, the long-term functional outcome after Traumatic Brain Injury (TBI) is assessed using the Glasgow Outcome Scale-Extended (GOSE), a global scale for functional outcomes, at 6 months after injury. GOSE 1–4 (death, persistent vegetative state, and severe disability) were regarded as unfavorable outcomes, while GOSE 5-8 (moderate disability, and good recovery) correspond to patients with favorable outcomes.
